# Vascular ring due to double aortic arch as a cause of stridor and dysphagia in infancy: a case report

**DOI:** 10.47487/apcyccv.v6i4.534

**Published:** 2025-12-29

**Authors:** Diego Davila-Flores, Christie Villasante-Villalta, Renee Montesinos-Segura, Juan Zúñiga-Meza, Henry Peralta-Santos, Luis Vera-Talledo

**Affiliations:** 1 Instituto Nacional Cardiovascular-INCOR, EsSalud, Lima, Perú. Instituto Nacional Cardiovascular-INCOR EsSalud Lima Perú; 2 Servicio de Cardiología Pediátrica. Instituto Nacional Cardiovascular-INCOR, EsSalud, Lima, Perú. Servicio de Cardiología Pediátrica Instituto Nacional Cardiovascular-INCOR EsSalud Lima Perú; 3 Servicio de Cirugía Cardiovascular Pediátrica. Instituto Nacional Cardiovascular-INCOR, EsSalud, Lima, Perú Servicio de Cirugía Cardiovascular Pediátrica Instituto Nacional Cardiovascular-INCOR EsSalud Lima Perú

**Keywords:** Vascular Rings, Double Aortic Arch, Dysphagia, Stridor, Pediatrics, Anillos Vasculares, Arco Aórtico Doble, Disfagia, Estridor, Pediatría

## Abstract

A double aortic arch is a rare congenital vascular anomaly with clinical significance in the pediatric population due to its potential to cause extrinsic compression of the trachea and esophagus. It should be suspected in infants with persistent respiratory and gastrointestinal symptoms refractory to conventional treatment. We report the case of a 2-year-7-month-old boy presenting with recurrent stridor, repeated respiratory infections, and progressive dysphagia. Cardiac CT angiography revealed a complete vascular ring causing tracheoesophageal compression. Dominance of the left aortic arch was identified, and surgical section and distal ligation of the non-dominant right arch were performed, without direct intervention on the tracheoesophageal structures. At the 12-month follow-up, there was complete resolution of symptoms and nutritional recovery. This case highlights the importance of maintaining high clinical suspicion and ensuring timely referral. Even when surgical correction is delayed, appropriate intervention can reverse symptoms and improve quality of life

## RESUMEN

El doble arco aórtico es una anomalía vascular congénita infrecuente, pero clínicamente significativa en la población pediátrica por su capacidad de generar compresión extrínseca de la tráquea y el esófago. Debe sospecharse en lactantes con síntomas respiratorios y digestivos persistentes, refractarios al tratamiento convencional. Presentamos el caso de un niño de 2 años y 7 meses con estridor recurrente, infecciones respiratorias a repetición y disfagia progresiva. La angiotomografía cardíaca reveló un anillo vascular completo con compresión traqueoesofágica. Se identificó dominancia del arco aórtico izquierdo, realizándose sección y ligadura quirúrgica distal del arco derecho no dominante, sin intervención directa sobre estructuras traqueoesofágicas. El seguimiento a los 12 meses mostró resolución total de los síntomas y recuperación nutricional. Este caso destaca la importancia de una sospecha clínica alta y de una derivación oportuna. Aún con corrección quirúrgica tardía, una intervención adecuada puede revertir el cuadro y mejorar la calidad de vida.

## Introduction

Congenital vascular rings are developmental anomalies of the aortic arch that encircle and compress the trachea and/or oesophagus, with an estimated incidence of 1 per 10,000 live births ^1^. Although they represent a small proportion of congenital heart defects, their clinical impact can be substantial, particularly in early childhood. Among these anomalies, the double aortic arch (DAA) is the most anatomically complete and the most clinically symptomatic form, accounting for 31-58% of all true vascular rings ^1^. Fetal echocardiography now enables prenatal suspicion of aortic arch anomalies through systematic assessment of the outflow tract and vessel course, facilitating postnatal diagnostic and therapeutic planning ^2^. Nonetheless, many cases go unrecognised until symptoms emerge during infancy, particularly in resource-limited settings where initial manifestations may be mistakenly attributed to common respiratory or gastrointestinal conditions ^3^.

We report the case of a 2-year-7-month-old infant presenting with resting stridor, recurrent respiratory infections, and progressive dysphagia, in whom a DAA with tracheo-oesophageal compression was diagnosed. This case underscores the importance of considering this anomaly in the differential diagnosis of persistent stridor and dysphagia in childhood, particularly in settings with limited access to specialised care, where delayed diagnosis can lead to respiratory complications and nutritional compromise. 

## Case report

A 2-year-7-month-old male patient residing in a high Andean region (3800 metres above sea level), with no relevant prenatal history, presented from 5 months of age with recurrent respiratory episodes characterised by cough, intermittent stridor, and dysphonia. These episodes were managed as bronchiolitis-like obstructive syndromes and pneumonias, treated with bronchodilators and corticosteroids, without sustained improvement. Given the persistence of symptoms, structural causes such as laryngomalacia, subglottic stenosis, and tracheomalacia were considered. Bronchofibroscopy revealed a 50% narrowing of the distal trachea, suggestive of extrinsic compression.

In the following months, respiratory episodes persisted, with baseline oxygen saturation remaining >90%. At 12 months of age, the patient developed progressive dysphagia for solid foods and was referred to paediatric gastroenterology. A contrast study of the oesophagus, stomach, and duodenum using a water-soluble agent showed no significant structural abnormalities.

**Figure 1:**
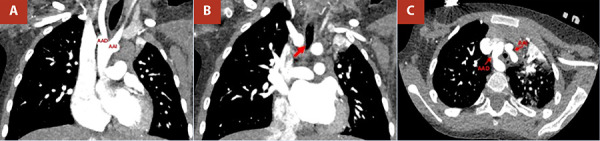
Computed tomography angiography of the chest. **(A)** Origin of the right and left aortic arches from the ascending aorta. **(B)** The right aortic arch produces extrinsic compression of the distal trachea (minimum diameter 3.4 mm) (red arrow). **(C)** Double right and left aortic arch forming a complete vascular ring around the trachea and oesophagus. RAA: right aortic arch. LAA: left aortic arch.

**Figure 2:**
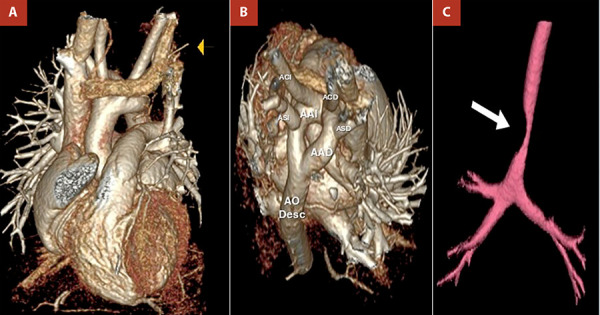
Three-dimensional (3D) reconstruction of chest CT angiography. **(A)** Anterior view showing the origin of the right and left aortic arches from the ascending aorta. **(B)** Right posterosuperior view demonstrating the aortic arches forming a complete vascular ring. **(C)** Severe extrinsic compression of the distal trachea. RAA: right aortic arch. LAA: left aortic arch. RCCA: right common carotid artery. LCCA: left common carotid artery. RSA: right subclavian artery. LSA: left subclavian artery. Desc. Ao.: descending aorta.

Given the persistence of respiratory and gastrointestinal symptoms and the absence of conclusive findings in previous studies, a cardiothoracic computed tomography (CT) angiography with three-dimensional reconstruction was requested. This examination revealed an aortic arch divided into two branches (right and left), with the common carotid and subclavian arteries arising from their respective ipsilateral arches (Figure 1). This anatomy corresponded to a complete vascular ring causing extrinsic compression of the distal trachea and oesophagus (Figure 2).

**Figure 3:**
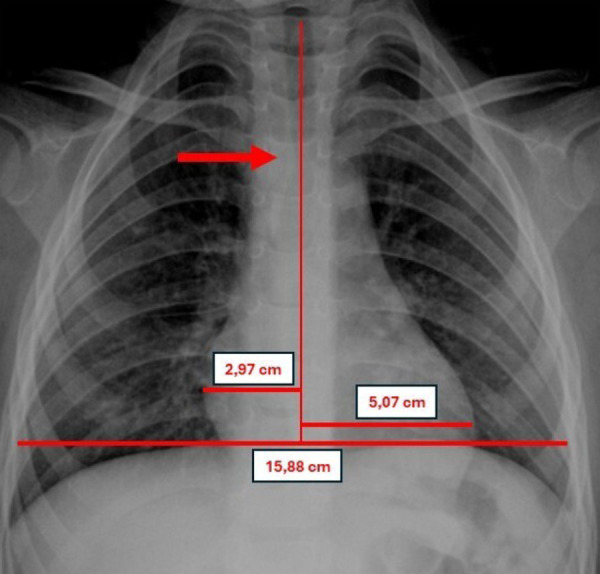
Anteroposterior chest radiograph showing preserved pulmonary vascularity, no evidence of cardiomegaly (cardiothoracic ratio: 0.50), and a notch in the mid-to-distal third of the trachea. CTR: cardiothoracic ratio.

The patient was referred late, 1 year and 7 months after symptom onset, to a national paediatric cardiovascular surgery centre, reflecting structural barriers within the health system that hinder timely access to specialised diagnosis and intervention in resource-limited settings. On initial evaluation, he presented with resting stridor, tachypnoea, oxygen saturation of 99%, and Z-scores of +0.6 for weight and -0.5 for height, suggesting mildly impaired nutritional status due to linear growth deceleration secondary to dysphagia. Chest radiography showed preserved pulmonary vascularity, no cardiomegaly, and a notch at the mid-to-distal third of the trachea (Figure 3).

Transthoracic echocardiography, using a longitudinal suprasternal view, demonstrated the presence of a DAA and ruled out associated congenital heart defects. Left-arch dominance was identified, and a right lateral thoracotomy was performed with ligation and division of the right arch distal to the origin of the ipsilateral vessels. No intervention on the trachea or oesophagus was required, as neither malacia nor structural lesions were observed.

Postoperative evolution was favourable, with no complications. The patient was discharged on day seven with acetylsalicylic acid (100 mg/day) for eight months as antithrombotic prophylaxis. At one-month follow-up, he showed clear clinical improvement with progressive resolution of dysphagia and stridor, as well as nutritional recovery (Z-scores: weight +0.2; height +1.1). 

At 12 months of follow-up, he remained asymptomatic, with no evidence of tracheo-oesophageal compression and an oxygen saturation of 99%.

## Discussion

During the first months of life, infants with a DAA typically present with respiratory symptoms such as stridor, chronic cough, or dysphonia, while dysphagia or feeding aversion tends to emerge progressively with the introduction of solid foods ^4^^,^^5^. The coexistence of both respiratory and gastrointestinal symptoms should prompt consideration of a structural compressive cause ^4^. In this case, the patient had persistent stridor and cough that were mistakenly treated with bronchodilators and corticosteroids from five months of age, and later developed dysphagia during the second year of life; a definitive diagnosis was established in the second year. Bronchoscopy revealed significant tracheal narrowing secondary to extrinsic compression. The contrast oesophagram was normal, highlighting a limitation of this technique: its sensitivity decreases with water-soluble contrasts or in cases of partial compression ^1^^,^^6^. Therefore, the absence of abnormalities does not rule out the diagnosis, and advanced imaging is required when clinical suspicion remains high ^1^^,^^6^.

Cardiac CT angiography confirmed the presence of a vascular ring, defined the anatomy and laterality of the aortic arch, and enabled surgical planning. The diagnostic modality of choice is currently considered for evaluating vascular rings due to their high spatial resolution and multiplanar capability, providing a detailed and non-invasive anatomical assessment ^7^^,^^8^. Doppler echocardiography is a useful initial tool for determining arch laterality ^1^^,^^6^, identifying aberrant subclavian arteries, and excluding associated congenital heart defects. However, its limitations in visualising extravascular structures constrain its diagnostic capacity; therefore, when clinical suspicion remains high, cardiac CT angiography should be performed ^1^^,^^6^.

Delayed diagnosis is common, with initial misdiagnoses such as asthma, recurrent respiratory infections, or laryngomalacia, largely due to the non-specific nature of symptoms and low clinical suspicion ^9^. This problem is exacerbated in settings with limited access to advanced imaging or specialised units, a situation frequently encountered in rural high-altitude regions or in health systems of resource-limited countries ^10^.

Surgical intervention is indicated in patients with vascular rings who present with severe respiratory or gastrointestinal symptoms, as well as in asymptomatic cases with evidence of tracheal or oesophageal compression involving ≥50% of the lumen ^11^^,^^12^. In the present case, the coexistence of persistent symptoms and a 60% extrinsic tracheal compression demonstrated by bronchoscopy justified surgical correction.

Treatment of a DAA consists of ligation and division of the non-dominant arch via sternotomy or lateral thoracotomy to relieve tracheo-oesophageal compression ^11^^,^^13^. In this patient, preoperative CT angiography identified the right arch as non-dominant; therefore, a right lateral thoracotomy with division and ligation was performed, without the need for additional intervention on the trachea or oesophagus, given the absence of residual structural abnormalities.

Although most cases of DAA are surgically treated within the first year of life, owing to the early recognition of compressive symptoms, the intervention in this report was performed at 2 years and 7 months of age. This delay reflects diagnostic limitations in resource-constrained settings. Clinical series report a median age at surgery between 10 and 15 months, and up to 13 months in specific cohorts (interquartile range [IQR]: 4-48 months) ^9^^,^^14^.

Observational studies, including a recent meta-analysis by Rato *et al.*, have shown that surgery for DAA is associated with high rates of symptom resolution, low morbidity, and minimal need for reintervention ^11^^,^^15^^,^^16^. Respiratory and gastrointestinal improvement may take weeks or even months, and in some cases up to a year, owing to tracheal and oesophageal remodelling ^4^^,^^6^. In the present case, recovery was progressive and complete, with resolution of stridor and dysphagia by three months after surgery, and the patient remained asymptomatic at 12-month follow-up.

Despite the lack of condition-specific studies, some reports have described the empirical use of prophylactic acetylsalicylic acid for 3 months following subclavian artery reimplantation, with no adverse events reported ^17^. Guidelines from the European Society of Cardiology support antiplatelet therapy in situations of elevated vascular risk, but they do not address congenital malformations of the aortic arch ^18^. Likewise, in complex congenital surgeries such as the Fontan procedure, aspirin (3-5 mg/kg per day) is recommended as standard prophylaxis. Although extrapolated, this therapeutic decision is grounded in practices described for cardiovascular interventions of comparable complexity and highlights the need for prospective studies to guide the safe and effective use, as well as the appropriate duration, of antithrombotic strategies in extracardiac malformations.

This case underscores the importance of maintaining a high index of suspicion when respiratory and gastrointestinal symptoms persist during childhood, as well as the need for timely referral. Although surgical correction was performed later than usual, the favourable outcome observed supports the notion that appropriate intervention, even outside the typical age window, can reverse the clinical course and substantially improve quality of life.

## References

[1] Chiu P, Zendejas B, Baird C (2023). Multidisciplinary approach to vascular rings and vascular-related aerodigestive compression a clinical practice review. Transl Pediatr.

[2] Bartsota M, Jowett V, Manuel D, Mortensen K, Wolfenden J, Marek J, Carvalho JS (2023). Double aortic arch implications of antenatal diagnosis, differential growth of arches during pregnancy, associated abnormalities and postnatal outcome. Ultrasound Obstet Gynecol.

[3] Porcaro F, Ciliberti P, Petreschi F, Secinaro A, Allegorico A, Coretti A, Cutrera R (2023). Long term respiratory morbidity in patients with vascular rings a review. Ital J Pediatr.

[4] Yoshimura N, Fukahara K, Yamashita A, Doi T, Yamashita S, Homma T (2020). Congenital vascular ring. Surg Today.

[5] Yesilbas O, Kus HD, Sik G, Citak A, Temur B, Yozgat CY (2020). Double aortic arch mimics the clinical characteristics of severe reactive airway disease in a pediatric patient. J Pediatr Intensive Care.

[6] Madira S, Orr WB, Rosenblum JM, Pitman R, Nguyen QT, Molter D (2025). Vascular rings - what has changed, and what do I need to know as a practitioner. Cardiol Young.

[7] Baz RO, Refi D, Scheau C, Axelerad A, Baz RA, Niscoveanu C (2024). CT angiography for aortic arch anomalies prevalence, diagnostic efficacy, and illustrative findings. Diagnostics (Basel).

[8] Priya S, Thomas R, Nagpal P, Sharma A, Steigner M (2018). Congenital anomalies of the aortic arch. Cardiovasc Diagn Ther.

[9] Ajdaa H, Carbonez K, Hubrechts J, Barrea C, de Beco G, Momeni M, Poncelet AJ (2024). Pediatric vascular ring outcomes for surgically repaired vs unoperated children: a single-center experience. J Thorac Dis.

[10] Fazlalizadeh H, Khan MS, Fox ER, Douglas PS, Adams D, Blaha MJ (2024). Closing the last mile gap in access to multimodality imaging in rural settings design of the imaging core of the Risk Underlying Rural Areas Longitudinal Study. Circ Cardiovasc Imaging.

[11] Gikandi A, Chiu P, Crilley N, Brown J, Cole L, Emani S (2024). Outcomes of patients undergoing surgery for complete vascular rings. J Am Coll Cardiol.

[12] Ruiz-Solano E, Mitchell M (2022). Rings and slings not such simple things. Curr Cardiol Rep.

[13] Harmandar B, Akcevin A, Aydemir NA, Onan IS, Calkavur T, Cicekcioglu H (2015). Surgical treatment of double aortic arch. Turk J Thorac Cardiovasc Surg.

[14] Yu D, Guo Z, You X, Peng W, Qi J, Sun J (2022). Long-term outcomes in children undergoing vascular ring division a multi-institution experience. Eur J Cardiothorac Surg.

[15] Swarnkar P, Speggiorin S, Austin BC, Nyman A, Salih C, Zidere V (2022). Contemporary surgical outcome and symptomatic relief following vascular ring surgery in children effect of prenatal diagnosis. Eur J Cardiothorac Surg.

[16] Rato J, Zidere V, Francois K, Boon M, Depypere A, Simpson JM (2023). Post-operative outcomes for vascular rings a systematic review and meta-analysis. J Pediatr Surg.

[17] Backer CL, Bharadwaj SN, Eltayeb OM, Forbess JM, Popescu AR, Mongé MC (2019). Double aortic arch with Kommerell diverticulum. Ann Thorac Surg.

[18] Aboyans V, Bauersachs R, Mazzolai L, Brodmann M, Palomares JFR, Debus S (2021). Antithrombotic therapies in aortic and peripheral arterial diseases in 2021: a consensus document from the ESC working group on aorta and peripheral vascular diseases, the ESC working group on thrombosis, and the ESC working group on cardiovascular pharmacotherapy. Eur Heart J.

